# Acute encephalomyelitis in a 52-year-old male post messenger ribonucleic acid severe acute respiratory syndrome coronavirus 2 vaccination: a case report

**DOI:** 10.1186/s13256-023-03831-2

**Published:** 2023-05-05

**Authors:** Pamela Lamisi Alebna, Muhammad Ahmad Shahid, Timothy Brannan, Ting Shen, Valentin Marian

**Affiliations:** 1 Internal Medicine, Rutgers/Robert Wood Johnson Barnabas Health Jersey City Medical Center, 355 Grand Street, Jersey City, NJ 07302 USA; 2 Neurology, Rutgers/Robert Wood, Johnson Barnabas Health Jersey City Medical Center, Jersey City, USA; 3 Pathology, Rutgers/Robert Wood Johnson Barnabas Health Jersey City Medical Center, Jersey City, USA; 4 Rheumatology, Rutgers/Robert Wood Johnson Barnabas Health Jersey City Medical Center, Jersey City, USA

**Keywords:** Vaccine adverse effects, Myelitis, ADEM, COVID vaccine

## Abstract

**Background:**

Acute disseminated encephalomyelitis is a well-known, but rare, side effect of some vaccines, or symptom following a febrile illness.

**Case:**

A 69-year-old, otherwise healthy Hispanic male presented with acute fever, confusion, and later progressive weakness after receiving the first dose of the mRNA-1273 (Moderna) severe acute respiratory syndrome coronavirus 2 vaccine. Considering the progressive deterioration of the patient, despite being on multiple immunosuppressive agents, a brain biopsy was obtained, which revealed nonspecific meningoencephalitis.

**Conclusion:**

In this case, we highlight the need for a regulatory framework to assist clinicians and patients with coverage of treatment for acute disseminated encephalomyelitis. The use of intravenous immunoglobulin in conjunction with glucocorticoids seems to be an effective treatment option.

## Introduction

With over 4.5 million lives lost to the coronavirus disease 2019 (COVID-19) pandemic globally [1], the development of COVID-19 vaccines, which have come to save millions of lives, has been timely indeed [2]. Reported adverse effects of vaccines have ranged from mild symptoms, such as fever, injection site reactions, and headaches [3], to more severe reactions such as thromboembolic phenomenon [4] and functional neurological disorders [5].

Acute disseminated encephalomyelitis (ADEM) is a well-known, but rare, side effect of some vaccines [6] or symptom following a febrile illness, especially in children. With no specific defining test, the diagnosis is usually made on the basis of medical history and exclusion of other plausible causes, such as Central Nervous System (CNS) infections, cerebrovascular accidents, malignancy, and autoimmune conditions. Treatment includes steroids and immunosuppressive agents.

In this report, we describe a case of a 69-year-old otherwise healthy male who presented with acute fever and confusion 4 days after receiving the first dose of the messenger RNA (mRNA)-1273 (Moderna) vaccine.

## Case

We present a case of a 69-year-old Hispanic male with no significant past medical history, who presented to the emergency department (ED) with complaints of fever and confusion for 3 weeks. His symptoms started 4 days after he received his first dose of the mRNA-1273 (Moderna) severe acute respiratory syndrome coronavirus 2 (SARS-CoV-2) vaccine. He had visited his primary medical doctor (PMD) a week prior to presentation. Initial work-up by his PMD revealed no abnormalities. He was treated symptomatically with acetaminophen and nonsteroidal antiinflammatory drugs. On the day of presentation, the patient woke up with a fever, excessive sweating, and an inability to answer questions appropriately. His family reported that his episodes of fever were associated with confusion, which would resolve after taking acetaminophen. He had no personal or family history of autoimmune disease.

On physical examination at the time of presentation, he was alert and oriented to time, place, and person. He had a fever of 102.6 °F, tachycardia of 115 beats per minute, and elevated blood pressure of 157/67 mmHg. He was responding to all questions appropriately. There was no neck stiffness or focal neurological signs. The rest of the motor and sensory examination was unremarkable.

**Fig. 1:**
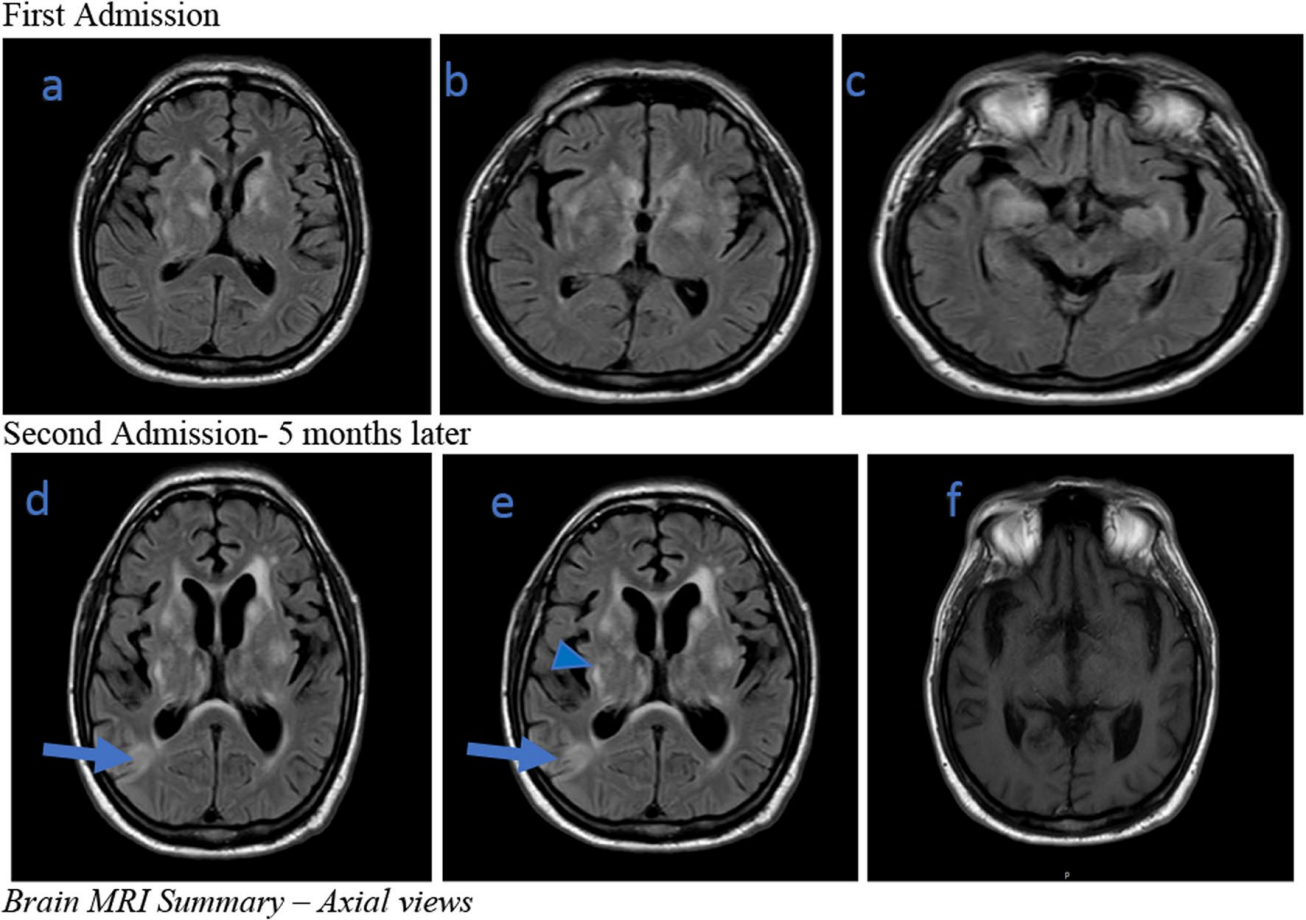
Brain MRI. Brain MRI summary—Axial views, Images **a**, **b**, **c** are images taken on initial presentation. Images **d**, **e**, **f** were taken at his second visit). Arrowhead: Generalized perivascular enhancement in the supratentorial brain during the second admission relative to first admission. There is faint non-specific enhancement in the periventricular regions and pachymeninges. There is progressive patchy prolongation in the white matter as well as deep gray matter structures. Blue arrows point to new infiltrates that were biopsied

Laboratory work-up showed an elevated erythrocyte sedimentation rate (ESR) of 44 mm per hour (reference range of 0–20 mm per hour). C reactive protein was normal at 0.5 mg per liter (reference range of < 3 mg per liter). Antinuclear antibodies, myeloperoxidase and proteinase-3, antineutrophil cytoplasmic antibody (ANCA), and complement proteins were normal. The remainder of the complete blood count, liver function test, and complete metabolic profile were unremarkable. A lumbar puncture was performed. The cerebrospinal fluid (CSF) was colorless with normal opening pressure. Analysis of the CSF revealed an elevated protein level of 119 mg per deciliter (reference range of 25–55 mg per deciliter), normal glucose concentration 52 mg per deciliter (reference range of 45–75 mg per deciliter), high leukocyte count of 300 per millimeter cube (reference range of 0–8 per millimeter cube), and neutrophilic pleocytosis with 72% polymorphonuclear cells. Other tests on the CSF included a nonreactive venereal disease research laboratory (VDRL) screen, negative oligoclonal bands, and negative JC virus Deoxyribonucleic acid (DNA) polymerase chain reaction (PCR). Serum protein electrophoresis was unremarkable. Magnetic resonance imaging (MRI) demonstrated increased abnormal fluid-attenuated inversion recovery (FLAIR) signals in the bilateral mesial temporal and anterior inferior frontal lobes, with small, scattered, ill-defined faint foci of enhancement (Fig. 1a–c). The possibility of viral encephalitis was a principal concern. Gram stain and fungal culture of the CSF were negative. CSF culture for fungi, bacteria, and viral panel were all negative. Other miscellaneous results, including results for urinalysis, parvovirus, and SARS-CoV-2, were all unremarkable.

On hospital days 2–3, the patient had not improved. He continued to spike a fever in the range of 102–103 °F, with no specific pattern noted, he had intermittent staring spells, and increased somnolence. At this point, most differentials that were considered at admission had been ruled out. Endocarditis was unlikely as cardiac echocardiography was negative for vegetations. Meningitis was unlikely given that the cerebrospinal fluid was sterile.

Considering the temporal relation of the patient receiving the SARS-CoV-2 vaccine and the onset of symptoms, there was a high suspicion that this presentation was due to vaccine-related acute disseminated encephalomyelitis. A multidisciplinary team including a rheumatologist, neurologist, and an infectious disease doctor made a collective decision to give the patient a trial of steroid therapy. He was commenced on glucocorticoids with mild improvement in symptoms; he completed 5 days of steroid therapy, and was discharged on prednisone 60 mg daily with a slow taper. Six weeks later, he was taking prednisone 40 mg per day with minimal improvement. At this juncture we decided to add intravenous immunoglobulin (IVIG) treatment 2 g per kg divided over 4 days. A significant and rapid improvement was noted since IVIG  was added. Unfortunately, due to off-label status of the IVIG  and insufficient insurance coverage, subsequent treatments were hampered and he experienced multiple setbacks.

### Readmission 5 months later

He presented 5 months after his initial episode with complaints of altered mental status, fever, and tremors. His wife reported that he was confused 2 days prior to receiving his last dose of intravenous IVIG. His baseline weakness had gotten worse and he was unable to ambulate, and began to have upper extremity tremors. Temperature checks at home had a maximum of 102 °F.

On examination, he was awake, did not engage in discussion, and required considerable effort to get him to follow commands. He had a fever of 101.5 °F. His laboratory findings were significant for leukocytosis of 15.4 × 10^9^ cells per liter, with differential neutrophilia. Urinalysis was suggestive of a urinary tract infection (UTI). Urine culture grew pan-sensitive *Citrobacter*. Analysis of the CSF in this admission revealed an elevated protein of 78 mg per deciliter (reference range of 25–55 mg per deciliter), normal glucose concentration 49 mg per deciliter (reference range of 45–75 mg per deciliter), a leukocyte count of 36 per millimeter cube (reference range of 0–8 per millimeter cube), lymphocyte count of 50%, and neutrophil count of 35%; thus, less inflammatory compared with CSF at his initial presentation.

The patient was started on antibiotic therapy with intravenous ceftriaxone for UTI, which resulted in some improvement in his mentation. However, he remained altered, only oriented to person.

**Fig. 2:**
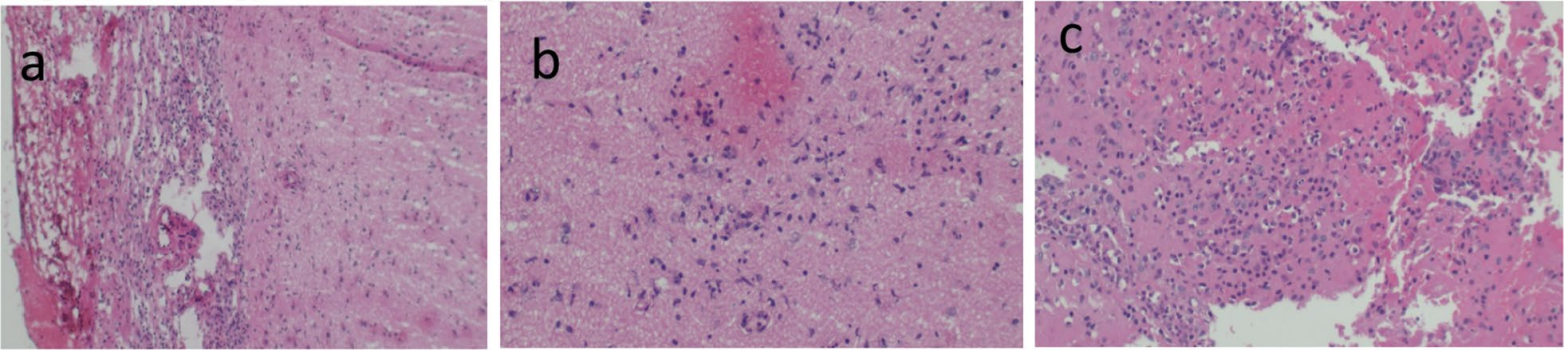
Histology of brain biopsy. **a** The leptomeninges are thickened and inflamed with many lymphocytes and some plasma cells. **b**, **c** The brain parenchyma with inflammatory infiltrates

MRI of the brain with gadolinium contrast material demonstrated worsening encephalitis (Fig. 1f). The interval changes in his MRI were evident of disease progression. Neurology service was consulted. At this point in management, postvaccine encephalitis remained high on the list of differentials; however, CSF vasculitis, CNS lymphoma, vector-borne viral encephalitis, or prion disease were all possibilities. Neurology recommended a brain biopsy of the right parietal cortex. With neurosurgery consultation and family consent, the right parietal brain biopsy was obtained. Histopathology demonstrated marked meningoencephalitis with associated reactive astrogliosis (Fig. 2a–c). Investigation for all other possible diagnoses were negative, the plausible diagnosis remained acute disseminated encephalomyelitis post SARS-CoV-2 mRNA vaccination.

## Discussion

Acute disseminated encephalomyelitis (ADEM) is an immune-mediated inflammation of the central nervous system that would usually follow a febrile illness, and in some cases vaccination [6]. Typical vaccines that have been associated with ADEM include rabies, measles, mumps, rubella, diphtheria, tetanus, polio, and Japanese B encephalitis [7]. Neurological symptoms are self-aborting, and in rare cases can lead to permanent disability. The most frequently observed symptoms include fever, headaches, or meningeal signs [7].

Review of the literature revealed four other reported cases of ADEM following Sinovac [8], another following Moderna vaccine [9], and two others related to the Pfizer vaccine [10]; a total of six reported cases in the Vaccine Adverse Event Reporting System (VAERS) [11]. Reported cases have varied deviations from the established criterion [12], suggesting that the presentations may not always be as expected.

Diagnosis can be quite challenging as there are several other central nervous system pathologies that present similarly. Diagnostic work-up to exclude infections, metabolic encephalopathy, CNS demyelinating diseases, and cerebrovascular accidents is important, which were all negative in our patient on both admissions.

Months had passed with no reasonable recovery, hence, considering the progressive deterioration of the patient and evidence of worsening disease on repeat brain imaging, we proceeded to obtain a brain biopsy. This was after extensive discussions with the neurosurgical team and the patient’s family. Brain biopsies are rarely obtained in clinical practice. In one other reported case of ADEM, a brain biopsy was obtained owing to diagnostic uncertainty [13]; histology revealed numerous inflammatory cells, absent fibrinoid necrosis or petechial hemorrhage, similar to findings in our case (Fig. 2).

There are no established evidence-based guidelines for the treatment of ADEM, treatment consists of immunosuppressive and anti-inflammatory agents [10]. He was initially treated with methylprednisolone, which was later switched to a tapering dose of prednisone. Each attempt to discontinue steroids led to worsening of symptoms. Intravenous immunoglobulin was then added to his treatment. The clinical progression of ADEM has not been formally studied, with some postulating that  a proportion of  patients eventually develop multiple sclerosis [10]. Most studies have focused on pediatric ADEM, which revealed that the spread, and location of the lesion on MRI predicts prognosis [9]. His family reported that after each episode of intravenous IVIG he was usually more functional, which eventually waned until his next session of IVIG—the infusions were three times per week.

It is important that, as we discuss the neurological adverse effects of SARS-CoV-2 vaccines, we add that vaccines are not in themselves the sole cause of these manifestations. Rather, they are idiosyncratic and a result of an individual’s own unique immune system’s response to the vaccine; hence, such a response could potentially be manifested with other triggers [10]. It is worth adding that encephalomyelitis has also been reported in persons with SARS-CoV-2 infection who have not received the vaccine [14].

## Conclusion

Immunization for SARS-CoV-2 with messenger RNA is generally safe, but rare severe side effects such as postimmunization encephalomyelitis could be seen. This is not a specific complication of SARS-CoV-2 mRNA vaccine, as it has been reported multiple times in the literature with other forms of immunization. A regulatory framework to assist clinicians and patients with coverage of treatment is imperative. Use of intravenous immunoglobulins  seems to be an effective treatment option when used in conjunction with glucocorticoids.

## Data Availability

Not applicable.

## Abbreviations

ED: Emergency department
ESR: Erythrocyte sedimentation rate
CSF: Cerebrospinal fluid
IVIG: Intravenous immunoglobulin
CNS: Central nervous system
